# Cyclic Fatigue of Dental NiTi Instruments after Plasma Nitriding

**DOI:** 10.3390/ma14092155

**Published:** 2021-04-23

**Authors:** Michal Bumbalek, Zdenek Joska, Zdenek Pokorny, Josef Sedlak, Jozef Majerik, Vlastimil Neumann, Karel Klima

**Affiliations:** 1 First Faculty of Medicine, Charles University Prague, 12108 Prague, Czech Republic; michalbumbalek@gmail.com (M.B.); karel.klima@lf1.cuni.cz (K.K.); 2 Faculty of Military Technology, University of Defence, 66210 Brno, Czech Republic; zdenek.pokorny@unob.cz (Z.P.); vlastimil.neumann@unob.cz (V.N.); 3 Faculty of Mechanical Engineering, Brno University of Technology, 61669 Brno, Czech Republic; sedlak@fme.vutbr.cz; 4 Faculty of Special Technology, Alexander Dubcek University of Trencin, 91101 Trencin, Slovakia; jozef.majerik@tnuni.sk

**Keywords:** nickel titanium, endodontic, plasma nitriding, cyclic fatigue, fracture

## Abstract

This study investigated the possibility of nitride NiTi instruments using low-temperature plasma nitriding technology in a standard industrial device. Changes in the properties and fatigue life of used NiTi instruments before and after low-temperature nitriding application were investigated and compared. Nontreated and two series of plasma-nitrided NiTi instruments, designed by Mtwo company with tip sizes of 10/.04 taper, 15/.05 taper, and 20/.06 taper, were experimentally tested in this study. All these instruments were used and discarded from clinical use. The instruments were tested in an artificial canal made of stainless steel with an inner diameter of 1.5 mm, a 60° angle of curvature, and a radius of curvature of 3 mm. A low-temperature plasma nitriding process was used for the surface treatment of dental files using two different processes: 550 °C for 20 h, and 470 °C for 4 h. The results proved that it is possible to nitride dental instruments made of NiTi with a low-temperature plasma nitriding process. Promising results were achieved in trial testing by NiTi instruments nitrided at a higher temperature. Plasma-nitrided files were found to have, in some cases, significantly higher values than nontreated files in terms of fatigue life. The results showed that the nitriding process offers promising possibilities for suitably modified surface properties and quality of surface layer of NiTi instruments. Within the limitations of the present study, the cyclic fatigue life of plasma-nitrided NiTi dental files can be increased using this surface technology.

## 1. Introduction

**Figure 1:**
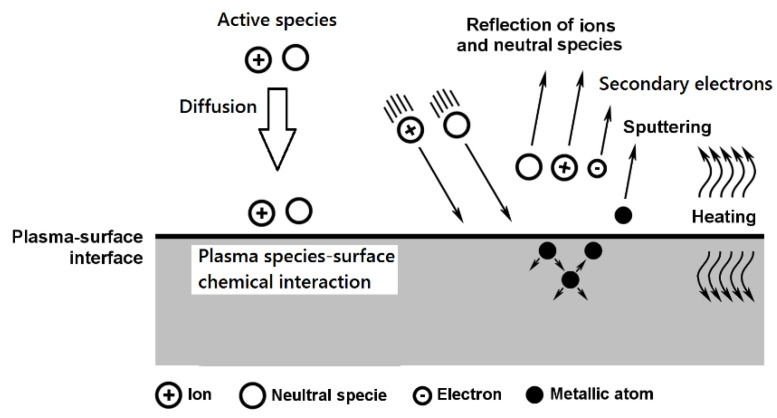
Illustration of events occurring during interaction between plasma and surface [31].

Nickel titanium (NiTi) alloys are widely used in biomedical devices due to their unique mechanical properties, such as superelasticity, shape memory, and good biocompatibility. However, the release of nickel ions from the surface of nitinol is a problem. In addition to implantology, endodontics is also a branch of medicine where these NiTi alloys are very often used [1,2]. The development of endodontic nickel titanium instruments improves the treatment possibilities for root canals of teeth. NiTi instruments adapt better to the shape of the root canal, making treatment faster and with better repeatability than stainless steel instrument treatment [2,3,4]. Although NiTi alloy has excellent mechanical properties, clinical practice has shown that the risk of breakage with these instruments is greater than with traditional stainless steel tools. One of the most common causes of tool damage is cyclic fatigue [4,5,6,7,8]. It has been found that the fatigue life is affected by many factors, the most important of which are the shape of the tool cross-section, type and quality of tool production, size and length of the tool, and tool movement in the root canal [9,10,11,12,13,14,15,16,17]. Mechanical surface treatment is among the most frequently used methods of surface treatment of NiTi alloys and includes the processes of grinding, sandblasting, and polishing [18,19]. Chemical heat treatment of NiTi alloys includes nitriding, ion implantation, and oxidation processes. These surface treatments offer very promising results, as they form a protective abrasion-resistant layer of TiN in the case of nitriding, or TiO_2_ in the case of oxidation, on the surface. Surface nitriding of NiTi alloys has been presented in many studies [20,21,22,23,24,25,26,27]. Ion implantation, gas nitriding, and powder nitriding are most often used to saturate the surface with nitrogen. These processes use a nitriding atmosphere of pure nitrogen [28,29,30] and a mixture of 90% nitrogen with 10% hydrogen [30]. All these processes take place at high temperatures in the range of from 800 °C to 1100 °C. The thickness of the layer varies depending on the nitriding time up to a thickness of 2 µm. Other types of nitriding include micropulse plasma nitriding. This type of process uses low temperatures in the nitriding process in the range of 300 °C to 580 °C. In this process, a mixture of nitrogen and hydrogen (N_2_ and H_2_) is most often used, where the molecules of these gases are cleaved and ionized in an electric field. Positive ions are accelerated towards the cathode, which is the surface of the nitrided components. Due to the sharp increase in the kinetic energy of the ions, the surface of the component is bombarded. Events occurring during the interaction between the plasma and the surface are shown in Figure 1.

Upon impact, heating converts kinetic energy into heat; the components are heated; and, at the same time, atoms of other elements, especially carbon, oxygen, and nitrogen, are ejected from the surface. By applying ionic sputtering before nitriding, it is possible to remove the surface layer of oxides [25], reduce the nickel concentration in the top layer of the NiTi alloy [32], and, thus, increase the formation of diffuse layers of titanium nitride and reduce the temperature of the nitriding process to about 500 °C.

The aims of this study were to verify whether it is possible to form nitrided layers with endodontic instruments, which have very small dimensions and a complicated shape compared to standard nitrided parts, and to verify the effect of formed layers on the cyclic life of endodontic instruments.

## 2. Materials and Methods

The experiments were based on the testing of real instruments used in dental treatment. NiTi instruments, designed by Mtwo (VDW, Munich, Germany) with tip sizes of 10/.04 taper, 15/.05 taper, and 20/.06 taper, were experimentally tested. These three types of instruments were chosen because they are most commonly used in clinical practice for the treatment of dental root canals. For these experiments, discarded tools from clinical practice were used.

### 2.1. Surface Treatment

Prior to the plasma nitriding process, NiTi instruments were collected and randomly divided into three groups: nontreatment (benchmark), plasma nitrided at 470 °C for 4 **h**, and plasma nitrided at 550 °C for 20 h. Before treatment, the instruments were submitted to ultrasonic cleaning in two stages of ten minutes each (first in acetone and second in isopropyl alcohol). For this experiment, industrial plasma nitriding equipment Rubig PN 60/60 (Rubig, Wels, Austria) was used. For plasma nitriding, two processes that are used as standards in nitriding steel parts were chosen. Since there is no information in the literature about the plasma nitriding of real endodontic instruments, two “boundary processes” that can be performed by the nitriding device were chosen. Process No. 1, consisting of a low nitriding temperature of 470 °C and a short nitriding time of 4 h, and process No. 2, consisting of a high nitriding temperature of 550 °C and a long nitriding time of 20 h, were selected. The working atmosphere of process was 100% N_2_. The concentration of N_2_ in the working atmosphere was necessary due to the diffusion process from the outer environment to NiTi materials.

Diffusion is dependent on temperature, pressure, duration, and suitable alloying elements in the nontreated material. These materials are very difficult to nitride, especially due to the low affinity of Ni for nitrogen [32]. Before the nitriding process, the sputtering process was included. The parameters of plasma sputtering and plasma nitriding are given in Table 1 and Table 2. The aims of the experiment were to compare the nitrided layers formed during these “boundary processes”, with the lowest possible temperature used for nitriding in this device used over a short time and the highest temperature used for nitriding used over a long time, and to verify whether they have a different effect on the fatigue life of endodontic instruments.

### 2.2. Chemical Composition

The chemical composition of the NiTi instruments was verified using the EDS method on a TESCAN MIRA 4 electron microscope. Line scan measurements of the chemical composition of the plasma-nitrided surface and the untreated surface were used. It is often stated in the literature [21,22] that the nickel content in the NiTi alloy is in the range of 48–52 wt %. During use, the chemical composition of the elements on the surface changes [23,24]; the nickel content decreases; and the content of titanium and other elements, such as nitrogen or oxygen, increases.

### 2.3. Sample Preparation for Evaluation of Nitrided Layer

The presence of a nitride layer in NiTi material was evaluated with the scanning electron microscopy method by using a TESCAN MIRA 4. The evaluation of the thickness of the nitride layer was performed on a cross-section. The samples of instruments were pressed into thermoplastic powder, subsequently ground, and, finally, polished. During the grinding process, the samples were checked step by step using optical microscopy. Finally, the samples were wet ground with sandpaper and polished using velvet with a diamond paste with grains 1 µm in size. The samples’ surface, thus prepared, was etched in an acid bath (HNO_3_: HF: CH_3_COOH 1:2:7). For the observation of the microstructure, the Olympus DSX 500i (Olympus, Tokyo, Japan) optical microscope with a magnification of 1000× was utilized. The evaluation of the fracture was performed on an SEM TESCAN MIRA 4 (Tescan, Brno, Czech Republic) without a special sample preparation process.

### 2.4. Cyclic Fatigue Test

**Figure 2:**
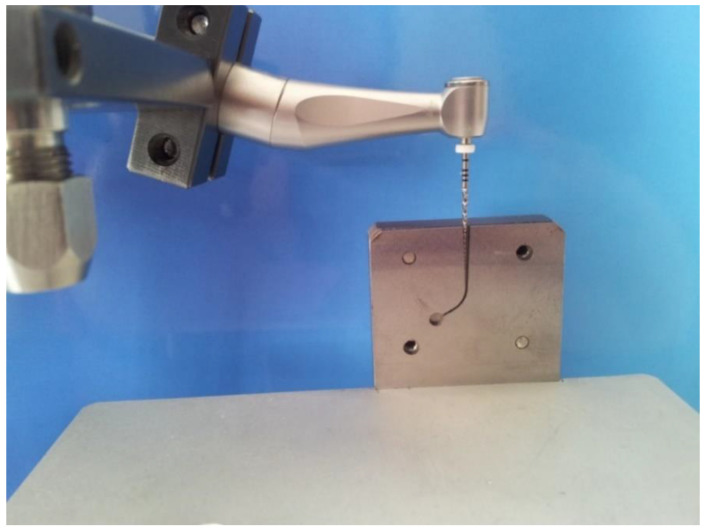
Testing stand of cyclic fatigue of NiTi instruments in artificial canal.

Cyclic fatigue testing in artificial canals was performed with an electric motor (Endo a class reciprocating LED, Medin, Czech Republic). The simulated canals were machined from a stainless steel block. The simulated canal was set as R 3 mm with an angle of 60° (Figure 2). The rotation speed was 190 rpm without torque control. The fracture time was recorded for each file in seconds and repeated ten times (n = 10). The corresponding fatigue life in the number of cycles was then accurately calculated.

## 3. Results and Discussion

### 3.1. Chemical Composition

**Figure 3:**
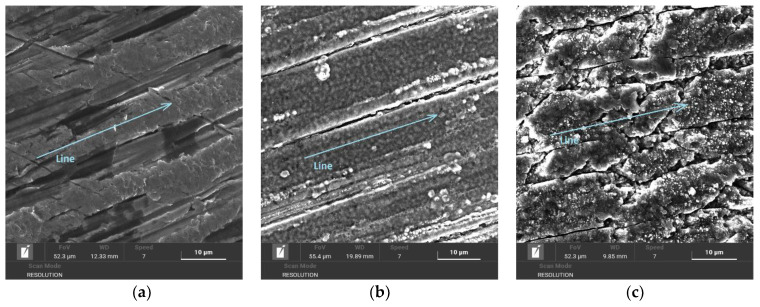
EDS line scan of untreated surface (**a**), plasma-nitrided surface after plasma nitriding at 470 °C and 4 h (**b**), plasma-nitrided surface after plasma nitriding at 550 °C and 20 h (**c**).

The chemical composition of dental NiTi instruments was verified using the EDS method due to a very small measurable area on the physical sample, as shown in Figure 3. The use of other typical spectroscopy testing methods was not possible.

The concentrations of Ni, Ti, and N elements were obtained via the EDS method using SEM TESCAN MIRA 4, and the results of the measurements are displayed in Table 3.

The content of elements measured by the analysis is in good agreement with the values measured in other studies [22,23,24]. The nickel content decreased in both plasma nitriding processes compared to the measurement performed on the untreated surface. The nitrogen content was higher in the plasma nitriding Process No. 2 and reached a value of 14 wt. %. It is well-known that alloys with a high concentration of Ni, Ti, Co, and Mn are very difficult to nitride [33]. The concentrations of Ni and Ti elements in this alloy are at a very high level (Table 3), and, therefore, the process of nitriding must be optimized in the future for supporting the diffusion process in this specific NiTi material during the process.

### 3.2. Assessment of Nitrided Layer

**Figure 4:**
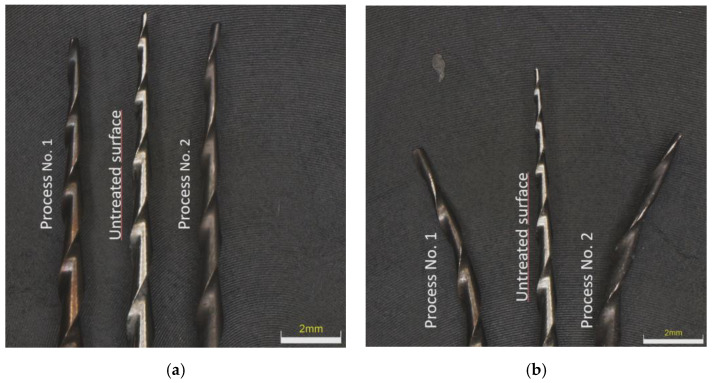
Macroscopic image of color change in the instrument with a tip size of 20/.06 taper prior and after plasma nitriding at 550 °C for 20 h (**a**) and rigid tip in the instrument with a tip size of 15/.05 taper after plasma nitriding at 550 °C for 20 h (**b**).

The surface documentation was provided using optical microscopy on an Olympus DSX 100 device. After plasma nitriding in both processes, the surface of the instruments changed color from metallic silver to dark bronze in Process No. 1 and to dark brown in Process No. 2, which indicates the probable presence of titanium nitrides, as seen in Figure 4a. Figure 4b shows an example of tool tips after the bending test of NiTi instruments. The untreated instrument showed a memory effect typical for these NiTi instruments, while, after both plasma nitriding processes, this memory effect was lost.

**Figure 5:**
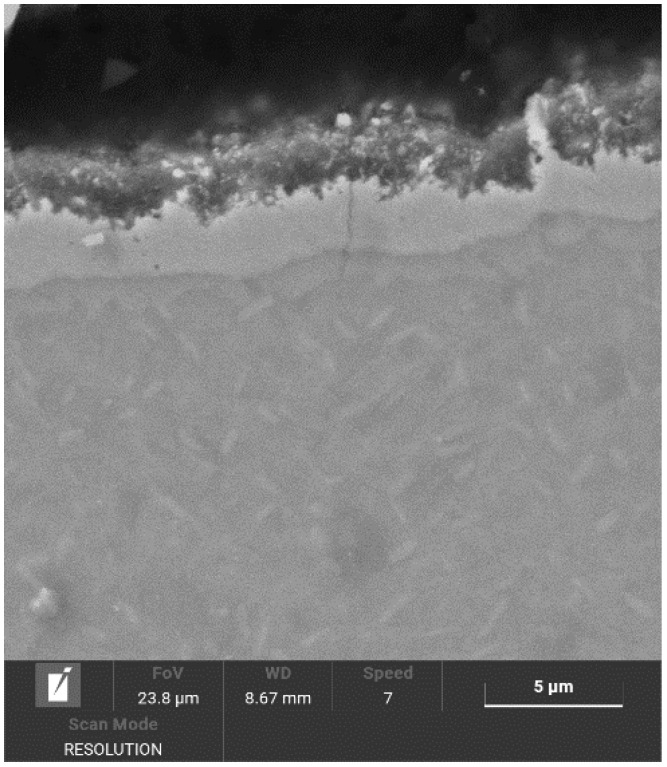
Nitrided layer on NiTi instrument with a tip size of 20/.06 taper after plasma nitriding at 470 °C and 4 h.

**Figure 6:**
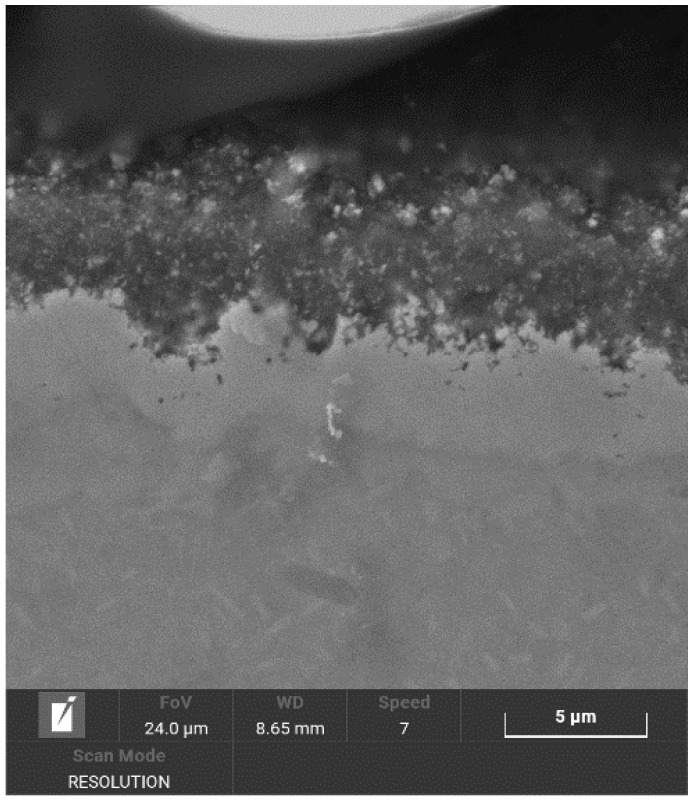
Nitrided layer on NiTi instrument with a tip size of 15/.05 taper after plasma nitriding at 550 °C for 20 h.

Figure 5 and Figure 6 show the structure of the nitrided layer after the processes, documented using SEM. The nitrided layers after both processes had a variable thickness: in the case of Process No. 1, the thickness of the nitrided layer reached 2–2.7 μm, and, in the case of Process No. 2, the thickness of the nitrided layer reached 3–3.8 µm.

**Figure 7:**
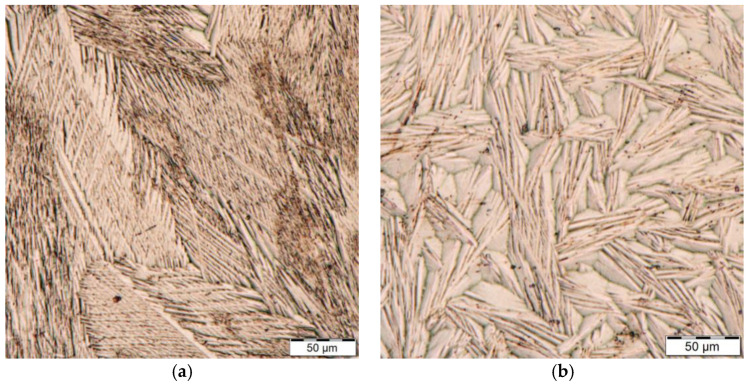
Microscopic image of different martensitic structures of instruments after plasma nitriding process 470 °C for 4 h (**a**) and plasma nitriding process 550 °C for 20 h. (**b**).

Figure 7a,b show the difference between the microstructure of NiTi after plasma nitriding. Figure 7a shows a fine martensitic structure, while Figure 7b shows a markedly coarse martensitic structure.

### 3.3. Cyclic Fatigue Test

**Figure 8:**
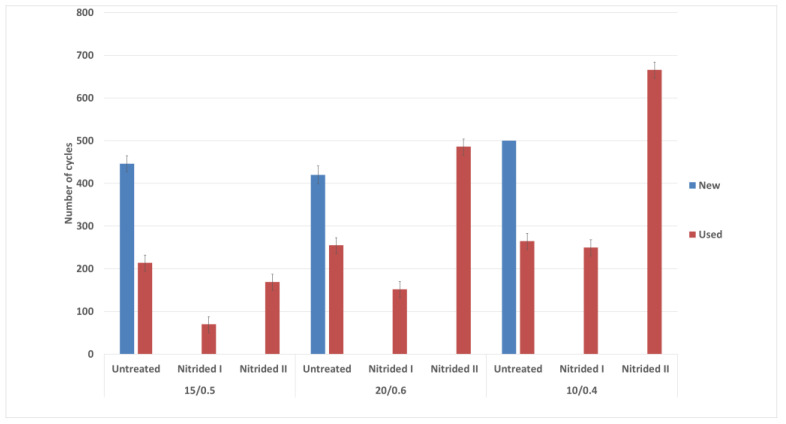
Results of cyclic fatigue test.

A simple cyclic fatigue test was performed to determine if the formed nitride layer affected the residual life of the instrument. In the cyclic fatigue test, there were significant differences in the cyclic fatigue of the untreated and nitrided instruments. Figure 8 Represents the comparison of individual tools after cyclic fatigue testing. The blue bars show new [34], unused instruments, which were also tested for comparison as the establishment of an initial state. For instruments with a tip size of 20/.06 taper and a tip size of 15/.05 taper, values were similar to the 420 cycles to fracture. For the tip size 10/.04 taper instruments, the number of cycles was 570 to breakage. For the instruments used (orange bar), the residual life in the raw state was measured. For instruments of all sizes, the results showed that instruments discarded from clinical use have half the service life of new instruments. For plasma-nitrided instruments after Process No. 1, the cyclic tool life of all three tool types was reduced. The most significant reduction occurred with the instrument with a tip size of 15/.05 taper. For plasma-nitrided tools in Process No. 2, the instrument with a tip size of 15/.05 taper had a small reduction in cyclic fatigue life compared to the instrument used. After Process No. 2, the cyclic fatigue life of the instrument with a tip size of 20/.06 taper increased, with the values being even higher than the new instrument. For the instrument with a tip size of 10/.04 taper, there was a marked increase in the residual cyclic life after Process No. 2 where the values reached 650 cycles compared to the 480 cycles reached by new instruments.

### 3.4. Fractographic Analysis

**Figure 9:**
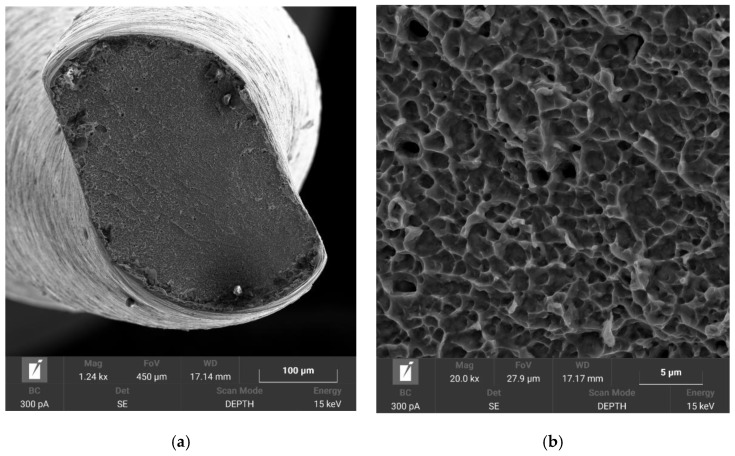
Macroscopic image of instrument with a tip size of 15/0.5 taper after plasma nitriding at 470 °C for 4 h (**a**) and detailed fracture surface (**b**).

**Figure 10:**
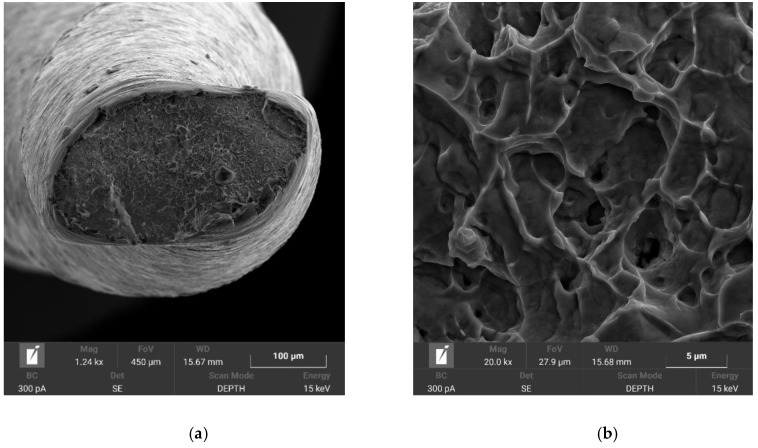
Macroscopic image of instrument with a tip size of 15/.05 taper after plasma nitriding at 550 °C for 20 h (**a**) and detailed fracture surface (**b**).

In both plasma nitriding processes, increased rigidity of the instruments was observed, where, during the experiment, it was evident from Figure 4b that the tool tip did not return to its original position. This can only be associated with the annealing of the NiTi base material during plasma nitriding, where the martensitic needles of the NiTi instrument coarsened. Fractographic analysis was performed with an electron microscope, TESCAN MIRA 4. In Figure 9 and Figure 10, the difference between the fractures of individual plasma-nitrided instruments is visible. The fractured character of the plasma-nitrided instruments can be seen to be changed and formed by a mix of ductile and brittle fractures. In plasma-nitrided tools, creek-shaped reliefs of the crack propagation process are visible. Furthermore, it can be seen that the microstructure of the fracture surface is different and is in good agreement with Figure 7a,b; in Process No. 1, the structure of the fracture surface is finer than that in Process No. 2. The coarsening of the instrument grain occurred because the nitriding process took place at a higher temperature of 550 °C and the cooling process in the nitriding device was slow. In contrast, nitriding at 470 °C did not cause such significant grain coarsening.

## 4. Conclusions

The nitrided layer in Process No. 1 reached a thickness of about 2–2.7 µm. In Process No. 2, there was an increase in nitrided layer thickness of 3–3.8 µm and in the size of martensitic needles in the base material. This was reflected in the nature of the tool fracture, where the shape and profile of the fracture surface was mixed with a large proportion of ductile fracture. In Process No. 1 there were finer martensitic needles in the base material, and the fracture surface did not show as high a proportion of ductile fracture. In both cases, with a more detailed observation of the fracture surfaces in all cases, the crack propagated along the grain boundaries. The results of cyclic fatigue were not unambiguous for nitrided instruments. For instruments with a tip size of 20/.06 taper and the instrument with a tip size of 10/.04 taper for Process No. 1, the number of cycles to fracture was reduced. Nitriding Process No. 2 had significantly better results with both tools, as the fracture speed was almost double that of the tools used, and even better than the new tools. For the instrument with a tip size of 15/.05 taper, the results were not unambiguous; for Process No. 1, there was a significant reduction in the number of cycles to fracture, and in Process No. 2, there was a partial reduction in the number of cycles to fracture. Based on these simple results, there is room for further investigation and optimization of plasma nitriding processes for endodontic instruments. By optimizing plasma sputtering, the composition of the nitriding atmosphere, voltage, and length of the micropulse discharge, it will be possible to create a suitable nitrided layer on endodontic instruments in the future.

Within the limitations of this study, nitrided endodontic instruments showed more promising physical and mechanical properties than conventional endodontic instruments. Future research should focus on new raw materials and thermomechanical processing procedures that can be used to further optimize the alloy microstructure in order to guarantee the reliability and safety of NiTi rotary instruments in clinical practice.

## Figures and Tables

**Table 1:** Parameters of plasma sputtering.

Plasma Sputtering					
Temperature (°C)	Time (h)	Gas Flow H_2_ (l·min^−1^)	Bias (V)	Pressure (Pa)	Pulse Length (µs)
450	0.5	8	800	80	100

**Table 2:** Parameters of plasma nitriding processes.

	Plasma Nitriding					
	Temperature (°C)	Time (h)	Gas Flow N_2_ (l·min^−1^)	Bias (V)	Pressure (Pa)	Pulse Length (µs)
Process No. 1	470	4	8	800	80	100
Process No. 2	550	20	8	800	80	100

**Table 3:** Chemical composition of untreated and plasma nitrided surfaces of NiTi instrument verified with EDS EDAX (wt. %).

	Ni	Ti	N	
	EDS			
Untreated surface	46	53	0	
Surface after Process No. 1	42	49	9	
Surface after Process No. 2	32	54	14	

## Data Availability

Data are contained within the article.
